# Clinical significance of kidney immune complex deposition in children with acute interstitial nephritis disease

**DOI:** 10.1080/0886022X.2023.2236234

**Published:** 2023-09-19

**Authors:** Pei Zhang, Xiao Yang, Xu He, Chun-Lin Gao, Zheng-Kun Xia

**Affiliations:** Department of Paediatrics, Jinling HospitalSchool of Medicine, Nanjing University, Nanjing, P.R. China

**Keywords:** Acute interstitial nephritis, immune complex deposition, renal pathology, prognosis

## Abstract

**Background:**

Acute interstitial nephritis (AIN) is a relatively rare cause of acute kidney injury (AKI) in children. Immune complex (IC) deposition was rare in renal pathology of AIN.

**Methods:**

Based on the status and position of IC deposition, a total of 78 children with AIN were divided into two groups: the non-IC group and IC group. IC group was further divided into two subgroups: intraglomerular (IG)-IC group and extraglomerular (EG)-IC group. To compare the clinical and histological features, renal outcomes between groups.

**Results:**

The IC deposition, IG-IC and EG-IC deposition were observed in 22 (28.21%), 12 (15.38%) and 10 (12.82%) children, respectively. The IC group demonstrated a higher frequency of AKI, higher level of Scr, urine N-acetyl-β-D-glucosidase (NAG) enzyme, retinol-binding protein (RBP), neutrophil gelatinase-associated lipocalin (NGAL), higher frequency of neutrophils, plasma cells and eosinophils infiltrate, higher scores of interstitial inflammation (i), total inflammation (ti) and interstitial edema, lower level of estimated glomerular filtration rate (eGFR) as compared to non-IC group (*p* < 0.05, *p* < 0.01). EG-IC deposition positively moderate correlated with levels of RBP, IG-IC deposition positively moderate correlated with plasma cell infiltrate, interstitial inflammation (i), total inflammation (ti) and interstitial edema. Interstitial inflammation, EG-IC deposition and interstitial edema were risk factors for AKD in AIN, and interstitial fibrosis/tubular atrophy (IF/TA) was a risk factor for CKD in children with AIN.

**Conclusion:**

IG-IC and EG-IC deposition positively correlated with severe clinical manifestations, glomerular and tubular injuries, and EG-IC deposition was risk factor for the progression of AIN in children.

## 1. Introduction

Acute interstitial nephritis (AIN) is an infrequent cause of acute kidney injury (AKI) characterized histologically by the presence of inflammatory infiltrates and oedema in the renal interstitium, usually associated with deterioration of renal function. AIN is an uncommon cause of AKI in the pediatric population, accounting for 0.87%∼15.7% of AKI in all children [1–2], and 27% in adults. AIN is usually caused by drugs, while other causes include autoimmune diseases [3] (systemic lupus erythematosus, Sjogren’s syndrome, sarcoidosis, IgG4-related immunoglobulin diseases), infections [4] (Legionella, Leptospira, Streptococcus, Corynebacterium, mycoplasma, hantavirus, measles, Epstein Barr virus, cytomegalovirus, HIV, etc.), herbal remedies and neoplastic conditions, etc. AIN is an inflammatory disease that affects the renal interstitium, which is characterized by the infiltration of T lymphocytes, monocytes, and eosinophils. Histology of renal biopsy usually shows inflammation and damage of the glomerulus and blood vessels as well as the interstitial structure of renal tubules [5].

In the previous literature, immune complex (IC) deposition was uncommon in AIN [6–7]. However, our results showed that IC deposition was observed in AIN, not only in the capillary loop and mesangial fields, tubulointerstitium was also the region of deposition. In this study, we aimed to explore the relationship between IC deposition and clinical manifestations, pathological changes, and prognosis in children with AIN, and to provide a theoretical basis for the diagnosis and treatment in children with AIN.

## 2. Materials and methods

### 2.1. Study design and patients

We performed a retrospective study on patients with biopsy-proven AIN from a single center between June 2007 and June 2022, at Jinling Hospital, School of Medicine, Nanjing University, Nanjing, China, to examine the clinico-pathological associations and the prognosis of children with AIN. All patients were <18 years old. Exclusion criteria were (1) glomerular diseases: anti-glomerular basement membrane nephritis, membranous nephropathy (MN), Henoch-Schonlein purpura nephritis (HSPN), IgA nephropathy (IgAN) and lupus nephritis (LN); (2) congenital and genetic kidney diseases; (3) antineutrophil cytoplasmicantibody-associated glomerulonephritis (AAGN); (4) tumor; (5) follow-up duration <12 months.

### 2.2. Laboratory findings

The medical records and pathologic data were reviewed and the following information at the time of renal biopsy as well as during follow-up was recorded: demographic characteristics, clinical features, hematology test, urine test and estimate glomerular filtration rate (eGFR). They included the following: gender and age. From blood samples the following data were also collected: white blood cell (WBC), erythrocyte sedimentation rate (ESR), procalcitonin (PCT), interleukin-6 (IL-6), serum creatinine (Scr), blood urea nitrogen (BUN), uric acid (UA) complement C3 and C4. Urine test items include N-acetyl-β-D-glucosidase (NAG) enzyme, retinol-binding protein (RBP) and neutrophil gelatinase-associated lipocalin (NGAL). The eGFR was calculated using the modified Schwartz formula [8]. Acute kidney injury (AKI), acute kidney disease (AKD) and chronic kidney disease (CKD) definitions were based on the Kidney Disease: Improving Global Outcomes (KDIGO) [9].

### 2.3. Renal histopathology

Two kidney pathologists evaluated all biopsies and were blinded to all clinical data and analyses. Renal biopsy specimens were examined using light microscopy, direct immunofluorescence (IF), electron microscopy (EM) and immunofluorescence staining techniques. Renal biopsy specimens were fixed in 2.5% paraformaldehyde for EM. Based on the status and position of IC deposition, the patients were divided into two groups: non-IC group, IC group, intraglomerular immune complex (IG-IC) group and extraglomerular immune complex (EG-IC) group. The IG-IC deposition was defined as deposits of immune complex observed in the glomerular capillary loop, mesangial fields and/or along the glomerular basement membrane (GBM) (either subendothelial, intramembranous, or subepithelial), and the EG-IC deposition was defined as deposits of immune complex were observed in TBM (intramembranous, interstitial, or epithelial side), interstitium, peritubular capillaries, arteries, and arterioles [10].

At least 10 proximal and non-proximal (distal or collecting ducts) tubular profiles for each patient were assessed with a focus on the appearance (quantity, electron density, and morphological pattern) and distribution of deposits. IC deposition was defined as IC (IgA, IgG, IgM, C3, C4, C1q) deposition ≥1 + [11]. The non-immune electron densities were excluded carefully in our observation; these densities were generally electron pale, possibly contained spherical structures, were located external to the TBM, and could be larger in size than the immune deposits.

Within a renal biopsy, infiltrates of neutrophils, eosinophils, plasma cells, and mononucleated cells (macrophages, lymphocytes) were quantified as a fraction of the area of total cortical inflammation [12]. Interstitial edema, tubular dilatation, epithelial simplification, brush border shedding, tubular vacuolization and casts were given a score between 0 and 4 as a percentage of the total cortical area of the biopsy (0: 0–1%, 1: >1-10%, 2: >10-25%, 3: >25-50%, and 4: > 50%) [13].

We observed the distribution of tubulointerstitial inflammation, interstitial edema and scored (score 1: < 25%, 2: 26-50%, and 3: > 50%). The degree of tubulitis were evaluated and scored (score 1: mild, 0-9 inflammatory cells/tubules, 2: moderate, 10-14 cells/tubules, and 3: severe, 15 or more cells/tubules in the most affected region) [14]. The total renal chronicity score including global/segmental glomerular sclerosis (score 0: < 10%, 1: 10-25%, 2: 26-50%, 3: >50%), interstitialfibrosis (score 0: <10%, 1: 10-25%, 2: 26-50%, 3: >50%), tubular atrophy (score 0: <10%, 1: 10-25%, 2: 26-50%, 3: >50%), and arteriosclerosis (score 0: intimal thickening < thickness of media, 1: intimal thickening ≥ thickness of media) [9]. Based on the individual lesions, each biopsy was evaluated according to the Banff scoring system, and the Banff score lesions include interstitial inflammation (i), tubulitis (t), arteritis (v), glomerulitis (g), interstitialfibrosis (ci), tubular atrophy (ct), arteriolar hyalinosis (ah), peritubular capillaritis (ptc), total inflammation (ti), inflammation in areas of IFTA (i-IFTA) and tubulitis in areas of IFTA (t-IFTA) [15].

### 2.4. Statistical analysis

All analyses were performed using SPSS (version 24.0, SPSS Inc, Chicago, IL, USA). Continuous variables are described as mean and standard deviation or median (interquartile range [IQR]) and categorized variables are described as percentages. Spearman correlation analyses were used to analyze correlations and shown by a heatmap reflecting mean values of Spearman’s ρ, asterisks indicate *p* < 0.05. The *r* > 0.5 was defined as a moderate correlation. Logistic regression analysis was used to analyze the risk factors of AKD and CKD in children with AIN. Variables with *p* > 0.1 were excluded by univariate Logistic regression analysis, and variables with *p* ≤ 0.1 were included in multivariate analysis. The prognosis was evaluated using Kaplan–Meier curves. *P* value of < 0.05 was considered statistically significant.

## 3. Results

### 3.1. Causative agents of AIN

**Figure 1.**
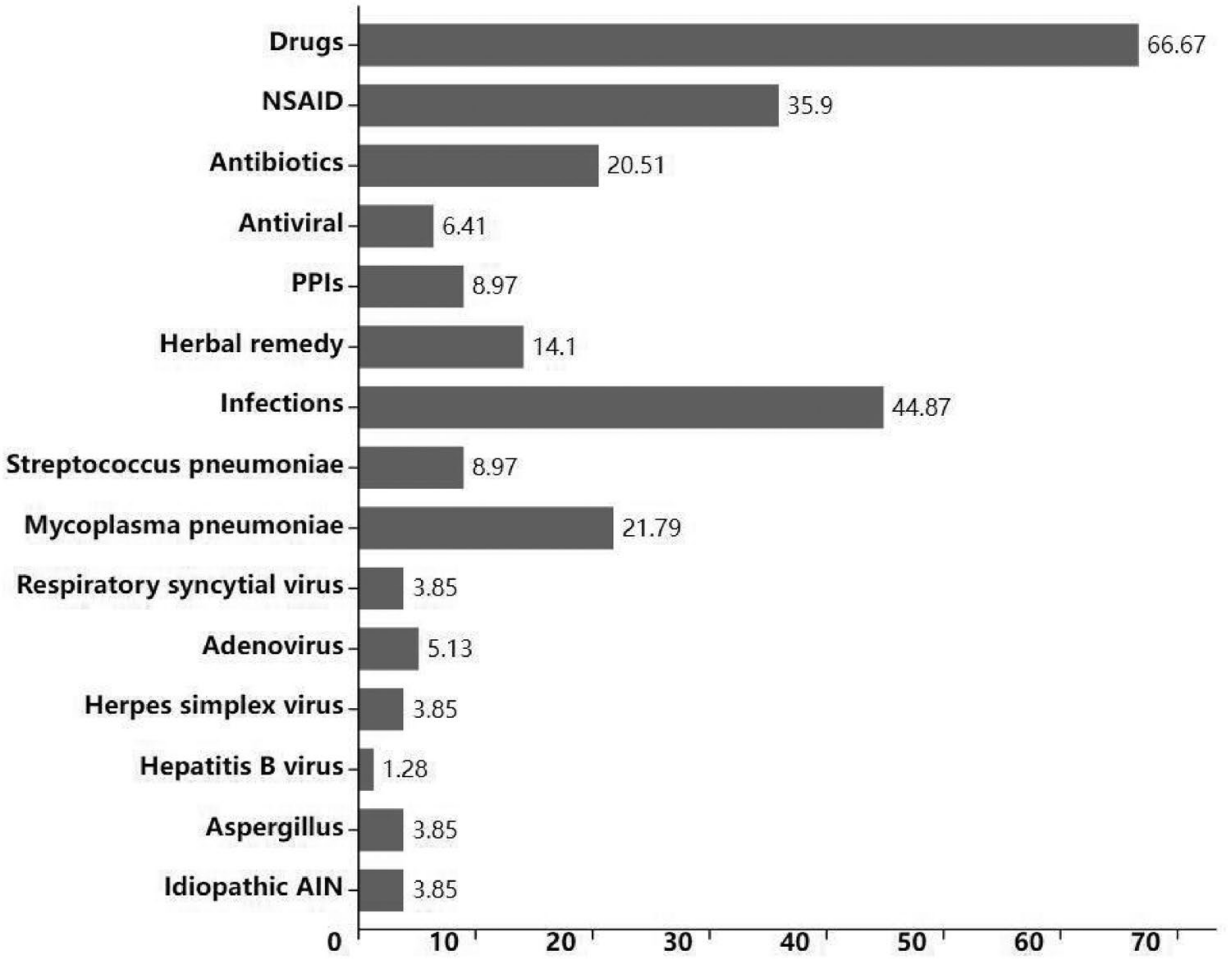
Etiology of children with AIN (%). NSAID: nonsteroidal antiinflammatory drug; PPIs: proton pump inhibitors; AIN: acute interstitial nephritis.

Seventy-eight children (53 males and 25 females) with a mean age of 12.35 ± 2.48 years old and a confirmed diagnosis of AIN were enrolled in the present study. Fifty-two cases (66.67%) were caused by drugs, and 35 cases (44.87%) were associated with infectious diseases. The etiology for 3 cases (3.85%) remained unclear. *Mycoplasma pneumoniae* (17, 21.79%) was the main cause of infectious diseases with AIN. NSAID and antimicrobials were identified as the most common causative drugs-induced TIN cases. NSAIDs accounted for 35.90% (28/78) of drug-induced cases, while 20.51% (15/78) of children received antibiotics treatment (Figure 1).

### 3.2. General clinical information

The presenting clinical symptoms include extrarenal and intrarenal features. Extrarenal syndrome showed fever in 25 (32.05%) children, rash in 6 (8.97%) children, eosinophilia in 5 (6.41%) children, abdominal pain in 30 (38.46%) children, nausea and vomiting in 44 (56.41%) children, anorexia in 16 (20.51%) children, joint pain in 6 (8.97%) children and hypertension in 4 (5.12%) children. Intrarenal syndrome included proteinuria in 52 (66.67%) children, hematuria in 57 (73.08%) children, leukocyturia in 37 (47.44%) children and oliguria/anuria in 26 (33.33%) children. Only 4 (5.12%) children had the triad of fever, rash, and eosinophilia concurrently. The IC group demonstrated a higher frequency of AKI, higher level of Scr, urine NAG enzyme, RBP, NGAL, and lower level of eGFR as compared to non-IC group (*p* < 0.05, *p* < 0.01). The percentage of RRT treatment, the incidence of AKD at 3 months and CKD at 12 months in IC group were higher than non-IC group (Table 1).

**Table 1.** Clinical and laboratory features, treatment and prognosis in children with AIN.

	AIN (*n* = 78)	non-IC group (*n* = 56)	IC group (*n* = 22)	*P* value*	IG-IC group (*n* = 12)	EG-IC group (*n* = 10)	*P* value^#^
Male sex, *n* (%)	53 (67.95)	39 (69.64)	14 (63.64)	0.609	6 (50.00)	8 (80.00)	0.145
Age in years	12.35 ± 2.48	11.87 ± 2.36	13.42 ± 3.11	0.536	12.65 ± 2.34	13.72 ± 2.55	0.522
Fever, *n* (%)	25 (32.05)	16 (28.57)	9 (40.91)	0.293	6 (50.00)	3 (33.33)	0.342
Rash, *n* (%)	6 (8.97)	4 (7.14)	2 (9.09)	0.771	2 (16.67)	0 (0.00)	–
Eosinophilia, *n* (%)	5 (6.41)	3 (5.36)	2 (9.09)	0.545	1 (8.33)	1 (10.00)	0.892
Clinical triad (fever, rash and eosinophilia), *n* (%)	4 (5.12)	3 (5.36)	1 (4.55)	0.884	1 (8.33)	0 (0.00)	–
Abdominal pain, *n* (%)	30 (38.46)	18 (32.14)	12 (54.55)	0.067	5 (41.67)	7 (70.00)	0.184
Nausea and vomiting, *n* (%)	44 (56.41)	30 (53.57)	14 (63.64)	0.420	8 (66.67)	6 (60.00)	0.746
Anorexia, *n* (%)	16 (20.51)	9 (16.07)	7 (31.82)	0.121	3 (25.00)	4 (40.00)	0.452
Joint pain, *n* (%)	6 (8.97)	3 (5.36)	3 (13.64)	0.217	3 (25.00)	0 (0.00)	–
Hypertension, *n* (%)	4 (5.12)	3 (5.36)	1 (4.55)	0.884	0 (0.00)	1 (10.00)	–
Proteinuria, *n* (%)	52 (66.67)	34 (60.71)	18 (81.82)	0.075	11 (91.67)	7 (70.00)	0.190
Hematuria, n (%)	57, (73.08)	40 (71.43)	17 (77.27)	0.601	10 (83.33)	7 (70.00)	0.457
Leukocyturia, *n* (%)	37, (47.44)	23 (41.07)	14 (63.64)	0.073	8 (55.56)	6 (60.00)	0.746
Oliguria/anuria, *n* (%)	26, (33.33)	16 (28.57)	10 (45.45)	0.155	7 (58.33)	3 (30.00)	0.184
AKI, *n* (%)	56, (71.79)	36 (64.29)	20 (90.91)	0.109	11(91.67)	9 (90.00)	0.892
WBC (×10^9^/L)	6.55 (4.75, 13.14)	6.34 (4.54, 14.27)	7.12 (5.43, 12.46)	0.480	6.78 (5.52, 11.35)	7.20 (5.15, 13.14)	0.305
ESR (mm/h)	36.56 (22.45, 54.25)	37.87 (20.26, 50.18)	33.19 (25.25, 57.83)	0.563	31.75 (23.06, 61.69)	35.65 (27.53, 55.47)	0.287
PCT (μg/L)	0.32 (0.04, 0.75)	0.35 (0.04, 0.65)	0.31 (0.04, 0.70)	0.417	0.29 (0.05, 0.75)	0.32 (0.03, 0.65)	0.294
IL-6 (ng/L)	18.95 (5.18, 48.55)	20.64 (4.97, 52.48)	16.76 (5.46, 44.15)	0.179	20.52 (4.85, 38.95)	15.05 (5.87, 51.36)	0.118
Scr (μmol/L)	176.25 (94.75, 368.50)	158.27 (75.48, 343.42)	196.43 (101.05, 380.47)	0.024	182.35 (110.58, 395.84)	204.85 (74.49, 357.75)	0.186
BUN (mmol/L)	17.25 (8.05, 26.75)	18.93 (7.76, 27.93)	14.95 (9.12, 23.43)	0.095	13.06 (9.56, 18.75)	16.27 (8.87, 25.05)	0.084
Serum UA (μmol/L)	376.43 ± 37.28	381.29 ± 38.75	363.26 ± 33.74	0.376	355.71 ± 33.74	384.55 ± 42.57	0.237
eGFR [ml/(min·1.73m^2^)]	66.75 (42.05, 84.75)	68.64 (48.70, 78.62)	54.37 (39.50, 84.42)	0.007	56.25 (42.76, 78.98)	51.65 (35.75, 88.32)	0.618
Serum complement C3 (g/L)	1.17 ± 0.21	1.19 ± 0.20	1.16 ± 0.18	0.346	1.14 ± 01.8	1.17 ± 0.19	0.537
Serum complement C4 (g/L)	0.24 ± 0.04	0.24 ± 0.03	0.25 ± 0.04	0.622	0.22 ± 0.02	0.26 ± 0.03	0.429
Urine NAG enzyme (U/g*cr)	26.52 (14.52, 51.45)	23.55 (14.75, 53.25)	32.05 (18.28, 64.75)	0.011	30.17 (20.36, 50.15)	34.25 (17.65, 57.58)	0.358
Urine RBP (mg/L)	0.73 (0.45, 4.28)	0.64 (0.42, 4.12)	0.79 (0.48, 5.15)	0.031	0.71 (0.39, 5.04)	0.67 (0.29, 5.24)	0.405
Urine NGAL (ug/L)	65.24 (36.25, 88.13)	56.53 (32.17, 76.45)	68.75 (38.94, 93.05)	0.009	66.43 (34.45, 97.25)	72.65 (41.76, 90.68)	0.413
Steroids, *n* (%)	54 (69.23)	37 (66.07)	17 (77.27)	0.336	9 (75.00)	8 (80.00)	0.782
RRT treatment, *n* (%)	15 (19.23)	6 (10.71)	9 (40.91)	0.002	5 (41.67)	4 (40.00)	0.937
AKD at 3 mo	25 (32.05)	14 (25.00)	11 (50.00)	0.033	5 (41.67)	6 (60.00)	0.392
CKD at 12 mo	15 (19.23)	7 (12.50)	8 (36.36)	0.016	4 (33.33)	4 (40.00)	0.746
ESRD	2 (2.56)	0 (0.00)	2 (9.09)	–	1 (8.33)	1 (10.00)	0.892

AIN: acute interstitial nephritis; IC: immune complex; IG: intraglomerular; EG: extraglomerular; AKI: acute kidney injury; WBC: white blood cell; ESR: erythrocyte sedimentation rate; PCT: procalcitonin; IL-6: interleukin-6; Scr: serum creatinine; BUN: blood urea nitrogen; UA: uric acid; eGFR: estimated glomerular filtration rate, NAG: N-acetyl-β-D-glucosidase; RBP: retinol-binding protein; NGAL: neutrophil gelatinase-associated lipocalin; RRT: renal replacement therapy; AKD: acute kidney disease; CKD: chronic kidney disease; ESRD: end-stage renal disease.

*p value non-IC group *VS* IC group; ^#^*p* value IG-IC group *VS* EG-IC group.

### 3.3. Pathologic characteristics

All biopsies were characterized by interstitial inflammatory cell infiltrate. The inflammatory cells were mainly neutrophils, mononuclear cells, plasma cells and eosinophils. Crescent was reported in 6.41% (5/78) of children. The average scores of interstitial inflammation (i), tubulitis (t), glomerulitis (g), total inflammation (ti) and interstitial edema at a high level. IC deposition was observed in 22 (28.21%) children, IgA in 10 (12.82%) children, IgG in 2 (2.56%) children, IgM in 12 (15.38%) children, C3 in 15 (19.23%) children and C1q in 1 (1.82%) child. Twelve (15.38%) children presented with IG-IC deposition, including 8 (10.26%) cases of IgA, 1 (1.28%) case of IgG, 8 (10.26%) cases of IgM, 10 (12.82%) cases of C3 and 1 (1.82%) case of C1q.Ten (12.82%) children presented with EG-IC deposition, including 2 (2.56%) cases of IgA, 1 (1.28%) case of IgG, 4 (5.13%) cases of IgM and 5 (6.41%) cases of C3. The frequency of neutrophils, plasma cells and eosinophils infiltrate, and the scores of interstitial inflammation (i), total inflammation (ti) and interstitial edema in IC group were higher than in the non-IC group (Table 2).

**Table 2.** Pathologic features in children with AIN.

	AIN (*n* = 78)	non-IC group (*n* = 56)	IC group (*n* = 22)	*P* value*	IG-IC group (*n* = 12)	EG-IC group (*n* = 10)	*P* value^#^
Interstitial inflammatory cell infiltrate	78 (100)	22 (100.00)	22 (100.00)	–	22 (100.00)	22 (100.00)	–
Neutrophils infiltrate, *n* (%)	45 (57.69)	27 (48.21)	18 (81.82)	0.007	10 (83.33)	8 (80.00)	0.840
Mononuclear cells infiltrate, *n* (%)	33 (42.31)	21 (37.50)	12 (54.55)	0.615	8 (66.67)	4 (40.00)	0.211
Plasma cells infiltrate, n (%)	37 (47.44)	22 (39.29)	15 (68.18)	0.021	9 (75.00)	6 (60.00)	0.452
Eosinophils infiltrate, *n* (%)	47 (60.26)	29 (51.79)	18 (81.82)	0.015	11 (50.00)	7 (70.00)	0.190
Crescent, *n* (%)	5 (6.41)	3 (5.36)	2 (9.09)	0.545	1 (8.33)	1 (10.00)	0.892
Interstitial inflammation (i)	1.68 ± 1.21	1.35 ± 0.75	1.86 ± 1.23	0.037	1.88 ± 1.42	1.65 ± 1.19	0.342
Tubulitis (t)	1.25 ± 1.06	1.26 ± 1.05	1.24 ± 1.03	0.742	1.24 ± 1.06	1.24 ± 1.08	0.816
Arteritis (v)	0.35 ± 0.28	0.36 ± 0.31	0.35 ± 0.24	0.728	0.34 ± 0.22	0.37 ± 0.25	0.624
Glomerulitis (g)	1.17 ± 0.94	1.18 ± 1.02	1.15 ± 0.76	0.297	1.15 ± 0.88	1.15 ± 0.69	0.775
Interstitial fibrosis (ci)	0.43 ± 0.37	0.43 ± 0.37	0.43 ± 0.39	0.686	0.46 ± 0.41	0.42 ± 0.39	0.527
Tubular atrophy (ct)	0.28 ± 0.16	0.27 ± 0.14	0.30 ± 0.18	0.327	0.31 ± 0.18	0.28 ± 0.16	0.532
Arteriolar hyalinosis (ah)	0.11 ± 0.08	0.11 ± 0.08	0.11 ± 0.07	0.713	0.12 ± 0.09	0.11 ± 0.08	0.426
Peritubular capillaritis (ptc)	0.08 (0.02, 0.12)	0.08 (0.03, 0.12)	0.08 (0.01, 0.11)	0.805	0.07 (0.02, 0.13)	0.09 (0.01, 0.09)	0.518
Total inflammation (ti)	1.84 ± 0.96	1.67 ± 0.85	1.93 ± 1.14	0.042	1.92 ± 1.13	1.93 ± 1.15	0.663
Inflammation in areas of IFTA (i-IF/TA)	0.13 (0.04, 0.22)	0.12 (0.03, 0.22)	0.15 (0.05, 0.21)	0.338	0.15 (0.05, 0.23)	0.15 (0.04, 0.19)	0.685
Tubulitis in areas of IFTA (t-IF/TA)	0.16 (0.06, 0.23)	0.14 (0.04, 0.23)	0.18 (0.08, 0.22)	0.186	0.19 (0.07, 0.21)	0.18 (0.09, 0.24)	0.594
Interstitial edema	2.35 ± 1.44	2.16 ± 1.19	2.52 ± 1.36	0.028	2.46 ± 1.20	2.65 ± 1.24	0.473
Epithelial simplification	1.02 (0.54, 1.65)	1.01 (0.52, 1.72)	1.05 (0.57, 1.54)	0.220	1.05 (0.55, 1.61)	1.05 (0.58, 1.52)	0.686
Tubular dilation	0.85 (0.32, 1.24)	0.84 (0.35, 1.20)	0.87 (0.31, 1.35)	0.304	0.88 (0.30, 1.37)	0.87 (0.33, 1.34)	0.564
Brush border shedding	0.65 (0.12, 1.26)	0.65 (0.14, 1.22)	0.65 (0.11, 1.28)	0.615	0.63 (0.14, 1.27)	0.66 (0.08, 1.30)	0.493
Tubular vacuolization	0.73 (0.35, 1.68)	0.72 (0.34, 1.66)	0.75 (0.38, 1.72)	0.365	0.75 (0.42, 1.76)	0.75 (0.35, 1.70)	0.718
Arteriosclerosis	0.08 (0.14, 1.26)	0.08 (0.12, 1.28)	0.07 (0.15, 1.22)	0.274	0.08 (0.13, 1.28)	0.06 (0.16, 1.19)	0.332

IF/TA intersititial fibrosis/tubular atrophy.

*p value non-IC group *VS* IC group; ^#^*p* value IG-IC group *VS* EG-IC group.

### 3.4. Correlations between immune complex deposition and clinical characteristics, laboratory markers and renal pathological changes

**Figure 2.**
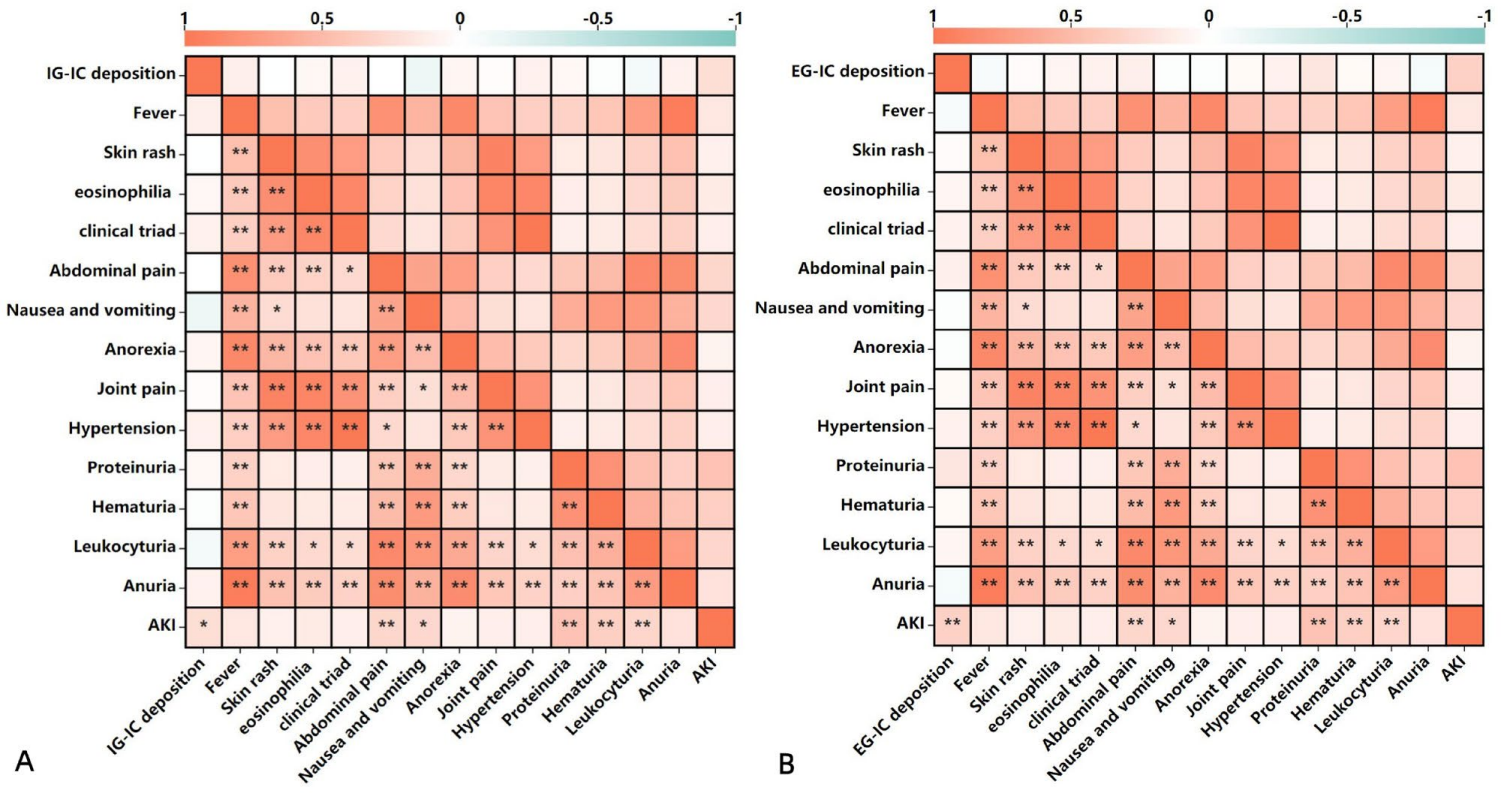
Correlation between immune complex deposition and clinical parameters. IC: immune complex; IG-IC: intraglomerular immune complex; EG-IC: extraglomerular immune complex; AKI: acute kidney injury.

**Figure 3.**
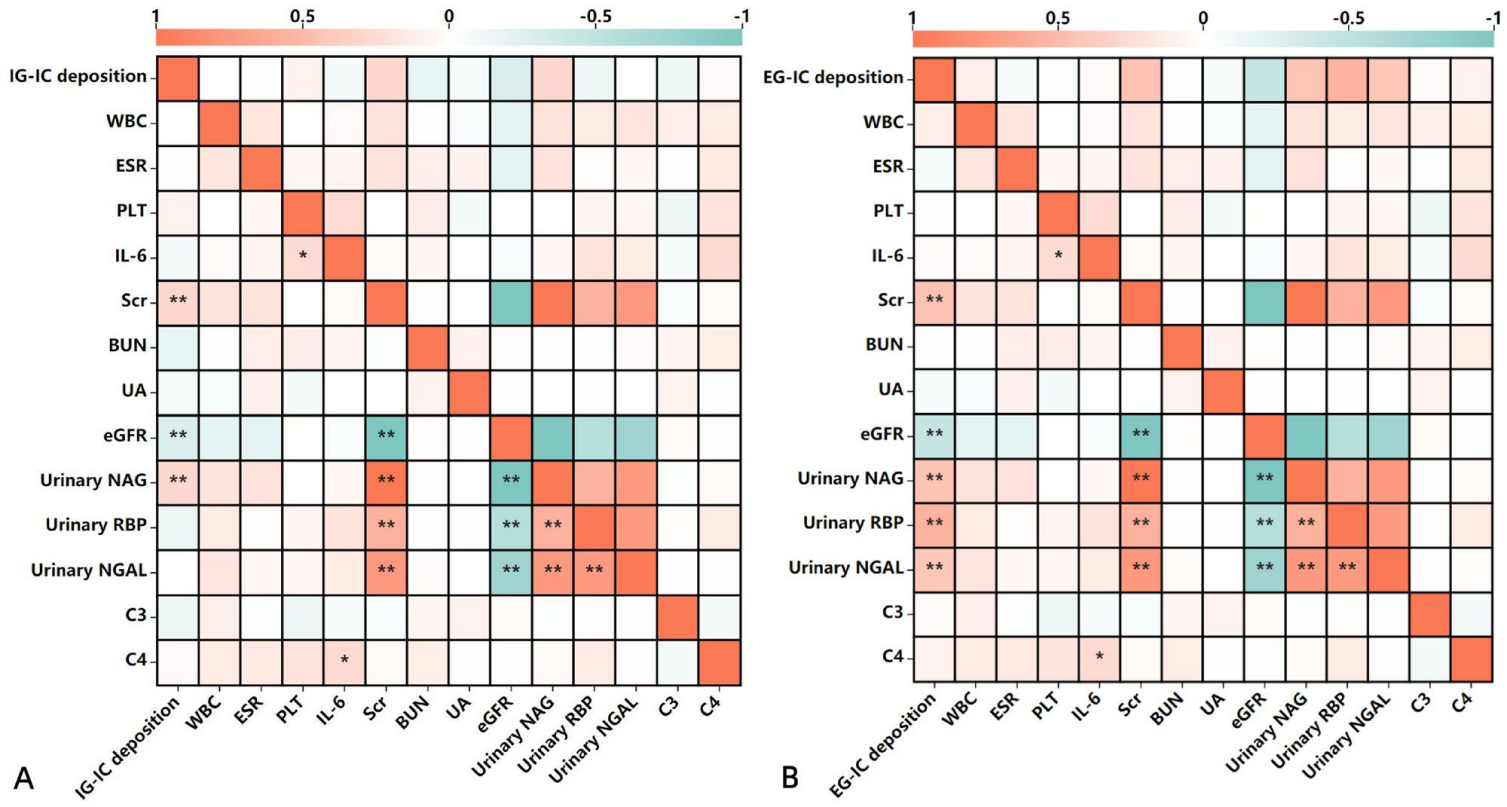
Correlation between immune complex deposition and laboratory findings. WBC: white blood cell; ESR: erythrocyte sedimentation rate; PCT: procalcitonin; IL-6: Interleukin-6; Scr: serum creatinine; BUN: blood urea nitrogen; UA: uric acid; eGFR: estimated glomerular filtration rate; NAG: N-acetyl-β-D-glucosidase; RBP: retinol-binding protein; NGAL: neutrophil gelatinase-associated lipocalin.

**Figure 4.**
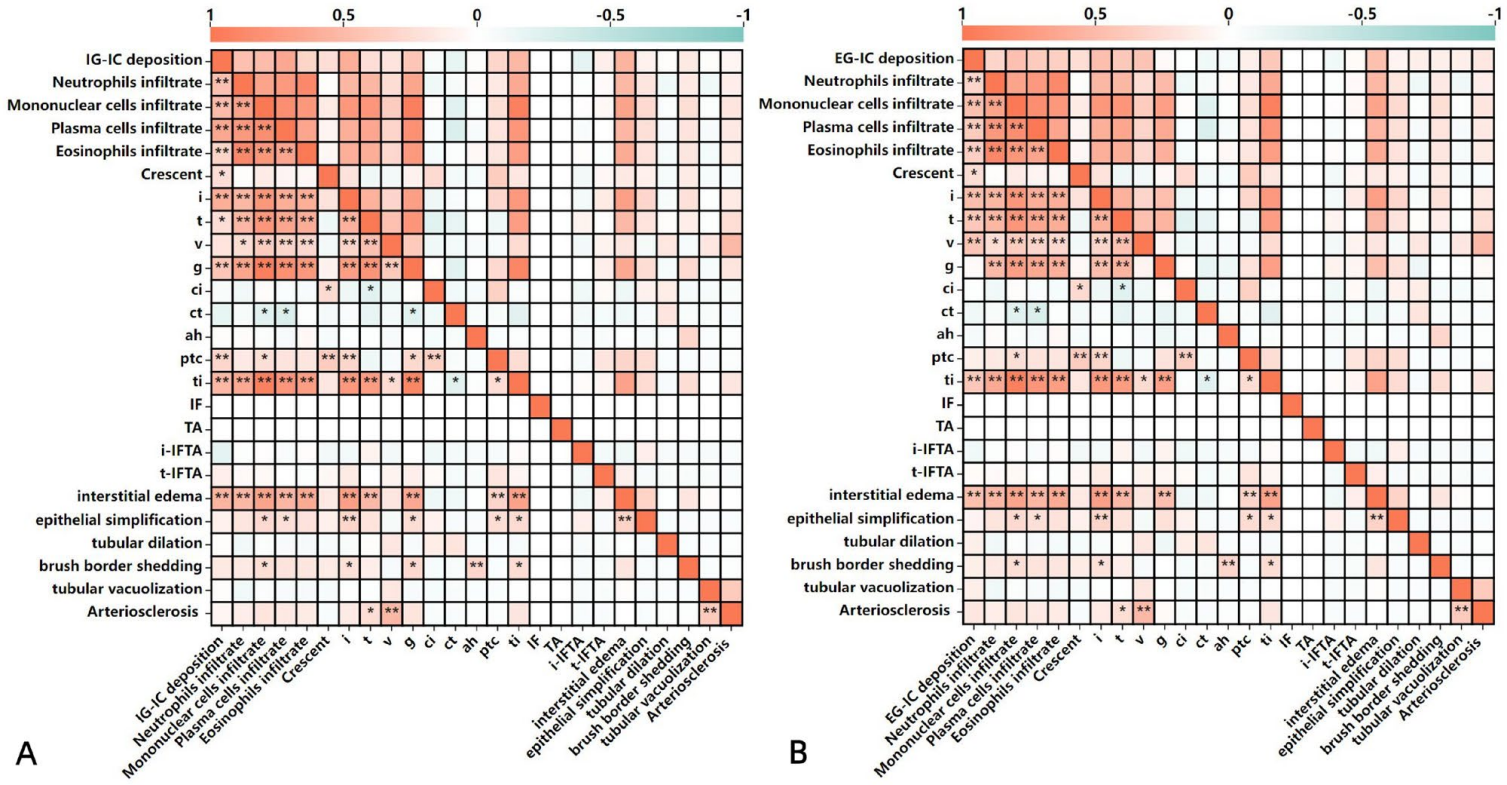
Correlation between immune complex deposition and pathological features. I: interstitial inflammation; t: tubulitis; v: arteritis; g: glomerulitis; ci: interstitialfibrosis; ct: tubular atrophy; ah: arteriolar hyalinosis; ptc: peritubular capillaritis; ti: total inflammation; i-IFTA: inflammation in areas of interstitial fibrosis/tubular atrophy; t-IFTA: tubulitis in areas of IFTA.

The results of the relationship between IC deposition and clinical and laboratory features were shown in Figure 2. IG-IC and EG-IC deposition positively correlated with AKI (*r* = 0.240, *p* = 0.003, *r* = 0.334, *p* = 0.002, respectively). Figure 3 showed that IG-IC deposition positively correlated with levels of Scr and urinary NAG (*r* = 0.300, *p* = 0.008, *r* = 0.292, *p* = 0.009, respectively), and negatively correlated with eGFR (r=-0.294, *p* = 0.009). EG-IC deposition positively correlated with levels of Scr, urinary NAG, RBP and NGAL (*r* = 0.445, *p* = 0.000, *r* = 0.442, *p* = 0.000, *r* = 0.564, *p* = 0.000, *r* = 0.417, *p* = 0.000, respectively), and negatively correlated with eGFR (*r*=–0.447, *p* = 0.000). The relationship between IC deposition and renal pathological lesions showed that IG-IC deposition positively correlated with neutrophils, mononuclear cells, plasma cells, and eosinophils infiltrate, crescent, interstitial inflammation (i), tubulitis (t), glomerulitis (g), peritubular capillaritis (ptc), total inflammation (ti) and interstitial edema (*r* = 0.468, *p* = 0.000, *r* = 0.490, *p* = 0.000, *r* = 0.647, *p* = 0.000, *r* = 0.317, *p* = 0.004, *r* = 0.245, *p* = 0.030, *r* = 0.600, *p* = 0.000, *r* = 0.244, *p* = 0.003, *r* = 0.426, *p* = 0.000, *r* = 0.309, *p* = 0.005, *r* = 0.530, *p* = 0.000, *r* = 0.551, *p* = 0.000, respectively), EG-IC deposition positively correlated with neutrophils, mononuclear cells, plasma cells, and eosinophils infiltrate, crescent, i, t, arteritis (v), ti and interstitial edema (*r* = 0.311, *p* = 0.006, *r* = 0.460, *p* = 0.000, *r* = 0.383, *p* = 0.000, *r* = 0.355, *p* = 0.001, *r* = 0.250, *p* = 0.028, *r* = 0.481, *p* = 0.000, *r* = 0.411, *p* = 0.000, *r* = 0.431, *p* = 0.000, *r* = 0.406, *p* = 0.000, *r* = 0.473, *p* = 0.000, respectively) (Figure 4). Above them, EG-IC deposition positively moderately correlated with levels of RBP, IG-IC deposition positively moderately correlated with plasma cell infiltrate, interstitial inflammation (i), total inflammation (ti) and interstitial edema.

### 3.5. Outcome and progression of AIN

After 3 months of follow-up, 42 children (53.85%) achieved complete recovery of kidney function, and 25 (32.05%) children achieved AKD. Fifteen (19.23%) children developed CKD by 12 months, and 2 children progressed to ESRD at the end of follow-up, both of the two children presented IC deposition. Finally, we analyzed the progression of AIN, and we observed that interstitial inflammation, EG-IC deposition and interstitial edema were risk factors for AKD in AIN, and interstitial fibrosis/tubular atrophy (IF/TA) was a risk factor for CKD in children with AIN (Table 3).

**Table 3.** Incidence of AKD and CKD in children with AIN (multivariate Logistic regression analysis).

	AKD		CKD 3-5 stage	
	*P*	HR(95% CI)	*P*	HR(95% CI)
Interstitial inflammation	0.019	15.534 (1.215∼ 24.183)	0.155	8.378 (1.538∼43.276)
IG-IC deposition	0.356	0.612 (0.297∼9.066)	0.108	4.559 (1.113∼9.357)
EG-IC deposition	0.024	0.759 (0.572∼ 1.536)	0.267	2.165 (0.243∼7.435)
Interstitial edema	0.036	3.582 (1.326 ∼7.879)	0.195	0.858 (0.126∼6.149)
IF/TA	0.572	0.813 (0.232∼2.719)	0.033	6.052 (1.621∼14.095)

AKD: acute kidney disease; CKD: chronic kidney disease; AIN: acute interstitial nephritis; IG-IC: intraglomerular immune complex; EG-IC: extraglomerular immune complex; IF/TA: interstitial fibrosis/tubular atrophy.

## 4. Discussion

AIN was described in 1898 by William Thomas Councilman, then pathologist-in-chief at the Brigham Hospital [16]. Renal histopathology in TIN is characterized by interstitial cellular infiltrates and edema, but vessels and glomeruli are typically spared. The inflammatory process may eventually lead to interstitial fibrosis and CKD.

The etiology of AIN can be classified as drugs, infections, autoimmune, systemic diseases, or idiopathic. Of this, drug-induced AIN accounts for over 2/3 of cases, followed by much lower frequencies of infection, idiopathic, tubulointerstitial nephritis and uveitis (TINU), and systemic autoimmune disease in decreasing order of frequency. TINU is usually considered an uncommon diagnosis, but relatively frequent in the pediatric population. In our cohort, there was no diagnosis of TINU, the reason may be that ophthalmological and renal lesions were not always simultaneous. The ophthalmological lesions may precede or follow the renal lesions [17]. And no etiology was found in three patients (3.85%) and the diagnosis was deemed to be idiopathic ATIN. The classic clinical triad of rash, fever, and eosinophilia is only present in about 15%-10% of cases [18]. The percentage of the clinical triad in our series of AIN children was 5.12%, abdominal pain, nausea and vomiting, proteinuria, hematuria, leukocyturia, oliguria/anuria and AKI were common clinical features and laboratory findings.

The results of immunofluorescence studies were negative in most patients. However, tubulointerstitial nephritis might be secondary to primary glomerulonephritis or occurs as a primary disease which may be due to IC deposition of antibodies directed against a structural component of the tubule, or due to direct toxic effects of poisons or drugs or infections. Tubular necrosis with deposits of immunoglobulins and/or complements was observed in renal biopsies [19]. In pathological conditions, there might be an alteration of the nature of the protein in the tubular or interstitial compartments, which might lead to the activation of complement, resulting in a pro-inflammatory response and eventually causing renal tubular degeneration and fibrosis [20]. It is generally believed that the initial event of AIN is the expression of endogenous nephritogenic antigens or exogenous antigens processed by tubular cells. According to research findings, immunization of rabbits or rats with Tamm-Horsfall protein or megalin induced an AIN [21], which suggested that these proteins might play a pathogenic role as endogenous antigens in the development of AIN, and other endogenous antigens, such as tubulointerstitial nephritis antigen, implicated in AIN had been identified as components of TBM [22].

TBM deposits could be present in patients with no or mild glomerular lesions, suggesting that TBM and glomerular IC formation might not be caused by the same mechanism [23]. TBM deposits could also be present in patients with no or mild tubulointerstitial lesions, suggesting that TBM deposits might occur before the appearance of severe tubulointerstitial injury [11], which also brought difficulties to the early diagnosis of TBM deposits and was the reason for the low rate of IC deposits. The mechanism of IG-IC deposition might be related to immune cell infiltrates, which lead to the activation of complement. Renal infiltration of plasma cells implies an alternative immunopathogenesis in which the antibody is produced locally by the infiltrating plasma cells and diffuses into the glomeruli and tubulointerstitium, also could into the TBM [24]. And immune deposits in the GBM and TBM were capable of activating serum complement, leading to immune-mediated glomerular and tubular injury [24]. Significantly, the mechanism of IG-IC and EG-IC deposition was different, the deposition of IC in glomeruli likely arises from a breach in systemic tolerance, in contrast, interstitial nephritis or tubular injury was associated with *in situ* tolerance diatheses [11]. Our study showed that there was no difference in clinical and pathologic characteristics between IG-IC group and EG-IC group, and the results of Spearman correlation analyses showed that IC deposition positively correlated with immune cell infiltrates, interstitial inflammation and edema, and did not correlate with C3 and C4 levels, the reason may be related to the time of complement testing and the cases of IC deposition. Literature reports about the IG - IC and EG - IC research was mainly on LN [10,23], therefore, the exploration of the difference between IG-IC and EG-IC in terms of the mechanisms of IC deposition in AIN was based on the large samples data.

All renal biopsies were examined by light microscopy (LM), We confirmed that IC deposition was not rare in AIN. The incidence of IG-IC and EG-IC deposition in our cohort was 15.38% and 12.82%, respectively. IG-IC and EG-IC deposition mainly positively correlated with acute renal tubular and kidney inflammatory lesions, and EG-IC deposition was a risk factor for AKD in AIN, which also confirmed that IC deposition might be an early lesion in AIN and suggested the importance of renal biopsy in an early stage of AIN. Furthermore, previous studies showed that IgG and C3 were the most common immune reactants on the TBM in IF observations [11,25], whereas, our study showed that IgM and C3 were the main deposit in AIN, indicating that IC deposition in AIN and autoimmune disease might not be caused by the same mechanism.

Fifty-four (69.23%) children were treated with corticosteroids, and fifteen (19.23%) children required renal replacement therapy (RRT) in our cohort. However, based on data on predominantly AIN, the evidence for routine steroid use has been inconclusive to date. The majority of AIN patients have a benign course and a complete or partial renal recovery. Discontinuation of the offending drug and infection clearance is the cornerstone of the treatment of AIN. However, in a small number of patients a poor recovery of kidney function is observed, and ESKD is not an uncommon consequence of drug and infection-induced AIN episodes. There were 2 children who progressed to ESRD at the end of follow-up in this cohort, and we observed that IF/TA was a risk factor for CKD in children with AIN.

The limitations of this study must be recognized. First, this study derived from the fact that was carried out in a single center. Second, children with mild AKI or atypical presentation of AIN may not have undergone renal biopsy and thus may be underrepresented in our findings.

In conclusion, the main causes of AIN in children are infection and drugs. IG-IC And EG-IC deposition positively correlated with severe clinical manifestations and glomerular and tubular injuries, and EG-IC deposition was a risk factor for AKD in AIN, which could be potential therapeutic targets. However, the specific mechanism of the IC deposition in AIN needs a large sample and multicenter in-depth study to clarify.

## Data Availability

All data generated or analyzed during this study are included in this published article.
